# Effect of intraperitoneal chemotherapy concentration on morbidity and survival

**DOI:** 10.1002/bjs5.50250

**Published:** 2020-01-16

**Authors:** F. M. K. Elekonawo, W. J. van Eden, W. Y. van der Plas, R. S. G. Ewalds, L. A. W. de Jong, A. J. A. Bremers, P. H. J. Hemmer, N. F. M. Kok, S. Kruijff, A. G. J. Aalbers, P. R. de Reuver

**Affiliations:** ^1^ Department of Surgery Radboud University Medical Centre Nijmegen Netherlands; ^2^ Department of Pharmacy Radboud University Medical Centre Nijmegen Netherlands; ^3^ Department of Surgical Oncology the Netherlands Cancer Institute Amsterdam Netherlands; ^4^ Department of Surgery University Medical Centre Groningen Groningen Netherlands

## Abstract

**Background:**

Selected patients with colorectal peritoneal metastases are treated with cytoreductive surgery (CRS) and hyperthermic intraperitoneal chemotherapy (HIPEC). The concentration of intraperitoneal chemotherapy reflects the administered dose and perfusate volume. The aim of this study was to calculate intraperitoneal chemotherapy concentration during HIPEC and see whether this was related to clinical outcomes.

**Methods:**

An observational multicentre study included consecutive patients with colorectal peritoneal metastases who were treated with CRS–HIPEC between 2010 and 2018 at three Dutch centres. Data were retrieved from prospectively developed databases. Chemotherapy dose and total circulating volumes of carrier solution were used to calculate chemotherapy concentrations. Postoperative complications, disease‐free and overall survival were correlated with intraoperative chemotherapy concentrations. Univariable and multivariable logistic regression, Cox regression and survival analyses were performed.

**Results:**

Of 320 patients, 220 received intraperitoneal mitomycin C (MMC) and 100 received oxaliplatin. Median perfusate volume for HIPEC was 5·0 (range 0·7–10·0) litres. Median intraperitoneal chemotherapy concentration was 13·3 (range 7·0–76·0) mg/l for MMC and 156·0 (91·9–377·6) mg/l in patients treated with oxaliplatin. Grade III or higher complications occurred in 75 patients (23·4 per cent). Median overall survival was 36·9 (i.q.r. 19·5–62·9) months. Intraperitoneal chemotherapy concentrations were not associated with postoperative complications or survival.

**Conclusion:**

CRS–HIPEC was performed with a wide variation in intraperitoneal chemotherapy concentrations that were not associated with complications or survival.

## Graphical abstract

Owing to differences in abdominal volume, intraperitoneal chemotherapy concentrations vary up to tenfold in patients treated with cytoreductive surgery and hyperthermic intraperitoneal chemotherapy. However, this variation was not associated with postoperative morbidity or survival.

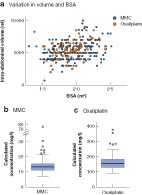

More attention needs to be given to drug concentrations

## Abstract (es)

**Antecedentes:**

Ciertos pacientes seleccionados con metástasis peritoneales de cáncer colorrectal (*peritoneal metastases*, PM) se tratan con cirugía citorreductora (*cytoreductive surgery*, CRS) y quimioterapia intraperitoneal hipertérmica (*hyperthermic intraperitoneal chemotherapy*, HIPEC). La concentración de quimioterapia intraperitoneal refleja la dosis administrada y el volumen perfundido. El objetivo de este estudio fue calcular la concentración de quimioterapia intraperitoneal durante HIPEC y evaluar si ello se relacionaba con los resultados clínicos.

**Métodos:**

Estudio observacional multicéntrico en el que se incluyeron pacientes consecutivos con PM de cáncer colorrectal que fueron tratados con CRS‐HIPEC entre 2010 y 2018 en tres centros holandeses. Se obtuvieron los datos a partir de bases de datos mantenidas de forma prospectiva. La dosis de quimioterapia y los volúmenes circulantes totales de solución de perfusión se usaron para calcular las concentraciones de quimioterapia. Las complicaciones postoperatorias y las supervivencias libre de enfermedad y global se correlacionaron con las concentraciones de quimioterapia intraoperatoria. Se realizaron regresiones logísticas univariable y multivariable, regresión de Cox y análisis de supervivencia.

**Resultados:**

De 320 pacientes, 220 recibieron mitomicina C intraperitoneal (MMC) y 100 oxaliplatino (OXA). El volumen medio de perfusión para HIPEC fue 5,0 L (rango 0,7‐10,0). La mediana de concentración intraperitoneal del agente quimioterápico fue de 13,3 mg/L (rango 7,0‐76,0) para MMC y 156,0 mg/L (rango 91,9 ‐ 377,6) en pacientes tratados con OXA. Las complicaciones de grado 3 o mayores ocurrieron en 23,4% (*n* = 75). La mediana de supervivencia global fue de 36,9 meses (rango intercuartílico 19,5‐62,9). Las concentraciones de quimioterapia intraperitoneal no se asociaron con las complicaciones postoperatorias ni con la supervivencia.

**Conclusión:**

La CRS‐HIPEC se realizó con una amplia variación en las concentraciones de quimioterapia intraperitoneal que no se asociaron con las complicaciones ni con la supervivencia.

## Introduction

Selected patients with colorectal peritoneal metastases are currently offered cytoreductive surgery combined with hyperthermic intraperitoneal chemotherapy (CRS–HIPEC). This results in improved median survival compared with systemic chemotherapy^1^, ^2^, ^3^.

Successful treatment of colorectal peritoneal metastases with CRS–HIPEC depends on several factors, including optimal patient selection and completeness of CRS^4^, ^5^. The concentration of the active agent used for intraperitoneal chemotherapy may also be important. The most widely used intraperitoneal chemotherapeutic drugs are mitomycin C (MMC), oxaliplatin and irinotecan^6^, any of which may be used with systemic therapies. Existing studies^7^, ^8^ have shown major differences regarding the intraperitoneal temperature, duration and perfusate volume. Intraperitoneal chemotherapy dosage is usually based on body surface area (BSA). Carrier solution volume has, however, received little attention. In the Netherlands, the carrier solution volume is not standardized. Volumes used reflect the remaining abdominal capacity after CRS. This itself is influenced by variations in tumour load, visceral resections and muscle tone of the abdominal wall. These variations inevitably result in different intraperitoneal chemotherapy concentrations being used in patients with similar BSA receiving similar drug doses.

Complication rates in patients treated with intraperitoneal MMC or oxaliplatin have been shown to be similar^9^, but the effects of higher concentrations of intraperitoneal chemotherapy have not been evaluated to see whether there is a relationship with increased occurrence of adverse events, or whether lower concentrations might be associated with worse survival. The aim of this study was to measure final intraperitoneal chemotherapy concentrations during the HIPEC and to evaluate their impact on complications and survival.

## Methods

This cohort study was performed in three tertiary institutes in the Netherlands: Radboud University Medical Centre, the Netherlands Cancer Institute (NCI) and University Medical Centre Groningen. Consecutive patients with colorectal peritoneal metastases who underwent primary CRS–HIPEC between 2010 and 2018 were eligible. Patients with appendiceal neoplasms other than adenocarcinoma and who were undergoing second and/or third HIPEC procedures were excluded. Prospectively developed databases of all patients treated with CRS–HIPEC were in place at all three centres. Before surgery, all patients were discussed in a multidisciplinary team meeting, involving surgeons, medical oncologists, radiologists, gastroenterologists and pathologists.

This study was performed in accordance with local medical ethical guidelines and collection of coded data was approved by the local medical ethical committee of Radboud University Medical Centre.

### Data collection and outcomes

Patient and treatment characteristics, along with operative details, details on the HIPEC procedure, histology findings, postoperative complications, disease‐free survival (DFS) and overall survival (OS) were recorded. The intraperitoneal chemotherapy concentration was calculated by dividing the administered chemotherapy dose by the total volume of instilled carrier solution.

Postoperative complications were scored according to the National Cancer Institute's Common Terminology Criteria of Adverse Events (v4.03)^10^ or the Clavien–Dindo classification^11^. DFS and OS were defined as the time from the date of operation to the date of disease recurrence or death, date of censoring or end of follow‐up. Patients were excluded if they had surgery less than 6 months before data analyses.

To assess the impact of intraperitoneal chemotherapy concentrations on secondary outcomes, patients were classified in three groups; for both MMC and oxaliplatin the different groups were based on the lowest 25 per cent, middle 50 per cent and highest 25 per cent intraperitoneal chemotherapy concentrations.

The Peritoneal Cancer Index (PCI)^12^ and Dutch Region Count^13^ were combined to create patient groups based on volume of disease categorized as limited, moderate or extensive peritoneal metastases. Patients with a PCI below 7 or a region count of 0–2 were placed in the lowest category. The moderate group consists of patients with a PCI of 7–20 or a region count of 3–5. Lastly, patients with a PCI above 20 or region count of 6–7 were placed in the group with extensive peritoneal metastases.

### Surgical procedure

During explorative laparotomy, the extent of peritoneal disease was scored according to the PCI^12^ and/or the Dutch Region Count^13^. Generally, when the PCI was 20 or less and/or the Region Count was 5 or less, the surgeons pursued complete cytoreduction. Completeness of cytoreduction score or the R score was used: CC0/R1 resection represents no visible macroscopic tumour nodules after cytoreduction; CC1/R2a resection represents tumour nodules smaller than 2.5 mm CC2/R2b resection represents tumour nodules of 2·5–25 mm; and CC3 represents tumour nodules greater than 25 mm^14^.

After exploratory laparotomy and CRS, HIPEC was performed, as described in detail elsewhere^2^. The open ‘coliseum technique’ was used to create a basin in the abdominal cavity. Two to four inflow catheters and two outflow catheters were used. The abdominal cavity was filled with a carrier solution (Dianeal® PD1.36; Baxter, Utrecht, the Netherlands) in the NCI, 5 per cent dextrose (Baxter) in Radboud University Medical Centre and University Medical Centre Groningen for oxaliplatin and 0·9 per cent sodium chloride for MMC until all peritoneal surfaces had been submerged. Chemotherapeutic drugs were added when the optimal temperature was steadily reached, as described below. Dosage of MMC or oxaliplatin was based on BSA, with a maximum BSA of 2 m^2^ for MMC. Patients received MMC or oxaliplatin according to institutional practice. In March 2014, Radboud University Medical Centre and the NCI switched standard MMC protocols to oxaliplatin.

For HIPEC with MMC, 35 mg/m^2^ heated to 41–43°C was administered for 90 min. Half of the total MMC dose was given at the start of the HIPEC procedure, a further one‐quarter 30 min after the start and the last one‐quarter of the total dose 60 min after the start. When oxaliplatin was used before HIPEC, intravenous leucovorin 20 mg/m^2^ was administered followed by 5‐fluorouracil 400 mg/m^2^. Thereafter, the carrier solution was heated to 43°C with oxaliplatin 460 mg/m^2^ added and perfused for 30 min. All patients were admitted to the ICU after surgery.

### Follow‐up

Biannual CT of the chest and abdomen was performed in the first 5 years after CRS–HIPEC, along with measurement of the serum tumour markers carcinoembryonic antigen, carbohydrate antigen (CA) 125 and CA‐19‐9. In the NCI, CT and serum tumour markers were performed annually after the first 2 years of biannual follow‐up. Recurrences and OS were registered.

### Statistical analysis

Mean and median values were analysed with Student's *t* test or the Mann–Whitney *U* test depending on distribution. Categorical variables were cross‐tabulated and significant differences identified using Fisher's exact test or the χ^2^ test as appropriate. Kaplan–Meier estimates of survival were calculated. OS was compared between groups with different intraperitoneal chemotherapy concentrations, using the log rank test. All tests performed were two‐sided, and *P* < 0·050 was considered statistically significant. Statistical analyses were performed with the SPSS® version 22.0 (IBM, Armonk, New York, USA).

Multivariable Cox regression analyses were performed with variables that were significant in univariable analysis or considered clinically relevant (tumour differentiation, N category, completeness of cytoreduction score and extent of disease).

## Results

A total of 320 patients underwent CRS–HIPEC and were included. Of these, 220 received intraperitoneal MMC and 100 received intraperitoneal oxaliplatin. Median follow‐up was 22·4 (range 0·1–122·6) months. Baseline characteristics of the two groups are described in *Table* 1.

**Table 1:** Patient, tumour and treatment characteristics

	All patients (*n* = 320)	MMC (*n* = 220)	Oxaliplatin (*n* = 100)	*P* †
**Patient characteristics**				
Age (years)*	59·7 ± 13·2	58·5 ± 14·0	62·4 ± 10·7	0·161‡
Sex ratio (M : F)	148 : 172	102 : 118	46 : 54	0·952
ASA fitness grade				0·655
≤ II	291 (90·9)	199 (90·5)	92 (92·0)	
> II	29 (9·1)	21 (9·5)	8 (8·0)	
Co‐morbidity				0·069
Cardiac	51 (15·9)	36 (16·4)	15 (15·0)	
Vascular	58 (18·1)	46 (20·9)	12 (12·0)	
Pulmonary	17 (5·3)	15 (6·8)	2 (2·0)	
Diabetic	28 (8·8)	20 (9·1)	8 (8·0)	
**Tumour characteristics**				
pT category				0·114
≤ pT3	128 (40·0)	95 (43·2)	33 (33·0)	
pT4	158 (49·4)	100 (45·5)	58 (58·0)	
pTx	34 (10·6)	25 (11·4)	9 (9·0)	
pN category				0·036
pN0	69 (21·6)	48 (21·8)	21 (21·0)	
pN1	100 (31·3)	71 (32·3)	29 (29·0)	
pN2	123 (38·4)	76 (34·5)	47 (47·0)	
pNx	28 (8·8)	25 (11·4)	3 (3·0)	
Time of diagnosis of peritoneal metastasis				0·128
Synchronous	169 (52·8)	115 (52·3)	54 (54·0)	
Metachronous	143 (44·7)	102 (46·4)	41 (41·0)	
Unknown	8 (2·5)	3 (1·4)	5 (5·0)	
Tumour location				0·184
Appendiceal adenocarcinoma	30 (9·4)	25 (11·4)	5 (5·0)	
Colon	253 (79·1)	171 (77·7)	82 (82·0)	
Rectum	37 (11·6)	24 (10·9)	13 (13·0)	
Differentiation grade				0·003
Good or moderate	184 (57·5)	124 (56·4)	60 (60·0)	
Poor	87 (27·2)	67 (30·5)	20 (20·0)	
Unknown	49 (15·3)	29 (13·2)	20 (20·0)	
Histology				0·020
Adenocarcinoma	188 (58·8)	117 (53·2)	71 (71·0)	
Mucinous adenocarcinoma	75 (23·4)	61 (27·7)	14 (14·0)	
SRCC	27 (8·4)	20 (9·1)	7 (7·0)	
Unknown	30 (9·4)	22 (10·0)	8 (8·0)	
**Treatment characteristics**				
Extent of peritoneal metastasis				0·002
Limited	115 (35·9)	69 (31·4)	46 (46·0)	
Moderate	143 (44·7)	99 (45·0)	44 (44·0)	
Extensive	21 (6·6)	14 (6·4)	7 (7·0)	
Unknown	41 (12·8)	38 (17·3)	3 (3·0)	
Completeness of cytoreduction				0·397
R1	303 (94·7)	207 (94·1)	96 (96·0)	
R2a	13 (4·1)	9 (4·1)	4 (4·0)	
R2b	4 (1·3)	4 (1·8)	0 (0)	
Clavien–Dindo complication grade	*n* = 319	*n* = 219	*n* = 100	0·013
No serious adverse events	133 (41·7)	84 (38·4)	49 (49·0)	
I–II	105 (32·9)	82 (37·4)	23 (23·0)	
III–IV	74 (23·2)	46 (21·0)	28 (28·0)	
V	7 (2·2)	7 (3·2)	0 (0)	
Unknown	1 (0·3)	1 (0·3)	0(0)	

Values in parentheses are percentages unless indicate otherwise

* values are mean(s.d.). MMC, mitomycin C; SRCC, signet ring cell carcinoma.

† χ^2^ or Fisher's exact test, except

‡ Student's *t* test.

**Figure 1:**
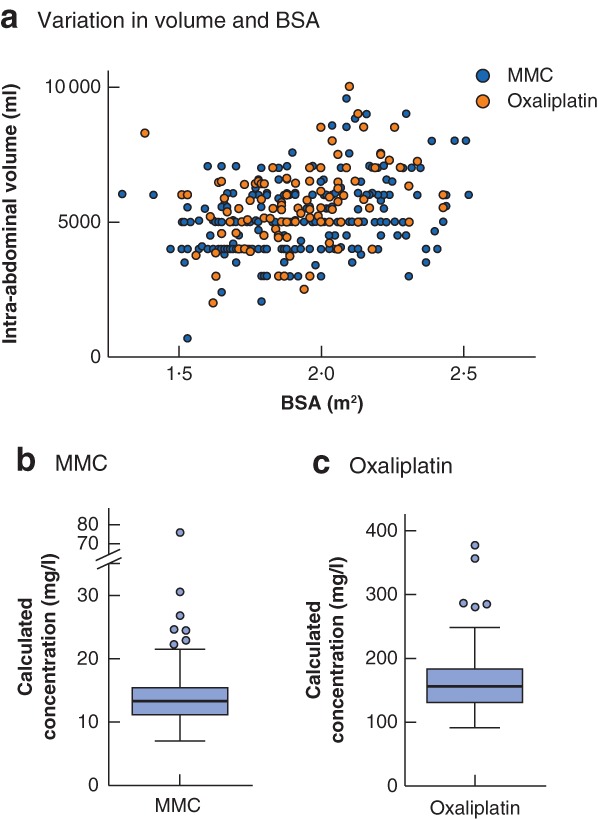
Details of intra‐abdominal volumes and body surface area, and calculated concentrations of mitomycin C and oxaliplatin **a** Scatterplot of intra‐abdominal volume and body surface area (BSA). Note the variation in intra‐abdominal volume at the same BSA. **b,c** Box‐and‐whisker plots of calculated concentrations of mitomycin C (MMC) and oxaliplatin. Median values, interquartile ranges and ranges (excluding outliers) are denoted by horizontal bars, boxes and error bars respectively.

Median BSA was 1·9 (range 1·3–2·5) m^2^. Median total dose of chemotherapy was 66·9 (range 35·0–89·1) mg for MMC and 877·0 (572·4–1060·0) mg for oxaliplatin. Median carrier solution volume was 5·0 (range 0·7–10·0) litres (*Fig*. 1 *a*). Median calculated intraperitoneal chemotherapy concentration was 13·3 (range 7·0–76·0) mg/l for MMC and 156·0 (91·9–377·6) mg/l for oxaliplatin (*Fig*. 1 *b,c*).

Grade III and above complications occurred in 81 of 320 patients (25·3 per cent) (*Table* 1). Median OS was 36·9 (i.q.r. 19·5–62·9) months for the overall cohort (36·9 (range 0·1–122·6) months for MMC and 29·5 (0·6–42·8) months for oxaliplatin; *P* = 0·516). Median DFS was 12·9 (range 0·1–107·4) and 13·1 (0·6–42·8) months in the MMC and oxaliplatin group respectively.

### Effects of concentration of intraperitoneal chemotherapy

*Table* 2 summarizes the different concentrations of MMC and oxaliplatin in the three stratified chemotherapy concentration groups and the association with postoperative surgical complications. Complication rates and grades were not significantly different between patients who received low, mid or high concentrations of intraperitoneal chemotherapy in either group (MMC: *P* = 0·492; oxaliplatin: *P* = 0·575).

**Table 2:** Postoperative complications and their relation to the calculated chemotherapy concentration used during hyperthermic intraperitoneal chemotherapy

		Clavien–Dindo complication grade		
Calculated concentration	*n*	0–II	III–IV	*P* *
**MMC (mg/l)**				0·492
Lower quartile (7·0–11·2)	55	45 (82)	10 (18)	
Mid range (11·2–15·4)	110	81 (73·6)	29 (26·4)	
Upper quartile (15·4–76·0)	55	41 (75)	14 (25)	
**Oxaliplatin (mg/l)**				0·575
Lower quartile (91·9–131·1)	25	16 (64)	9 (36)	
Mid range (131·3–184·0)	49	37 (76)	12 (24)	
Upper quartile (184·0–377·6)	26	19 (73)	7 (27)	

Values in parentheses are percentages. MMC, mitomycin C.

* χ^2^ or Fisher's exact test.

**Figure 2:**
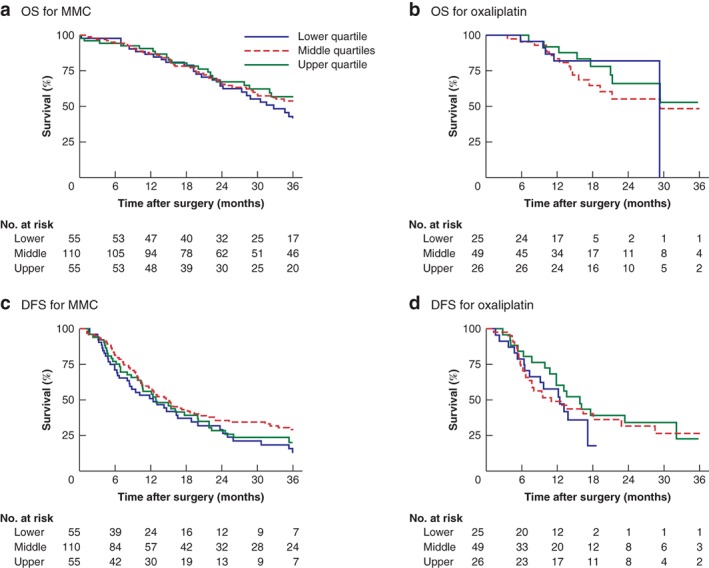
Kaplan–Meier analysis of overall and disease‐free survival for different intraperitoneal chemotherapy concentrations **a,c** Overall (OS) and disease‐free (DFS) survival in patients receiving mitomycin C (MMC) and **b,d** OS and DFS in patients receiving oxaliplatin, according to lower quartile, mid range and upper quartile intraperitoneal chemotherapy concentration. **a** *P* = 0·671, **b** *P* = 0·703, **c** *P* = 0·170, **d** *P* = 0·624 (log rank test).

*Fig*. 2 illustrates OS and DFS for MMC and oxaliplatin per stratified chemotherapy concentration group (lower quartile, mid range and upper quartile). No significant association was observed between any of the concentration groups and OS or DFS.

**Figure 3:**
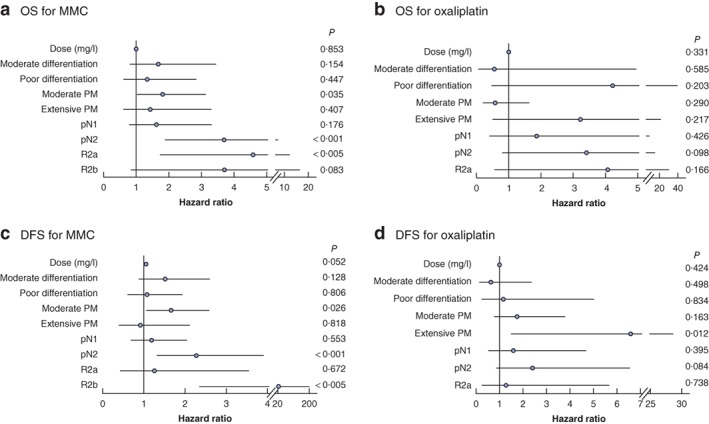
Multivariable regression analysis of overall and disease‐free survival in patients receiving mitomycin C or oxaliplatin **a,c** Overall (OS) and disease‐free (DFS) survival in patients receiving mitomycin C (MMC) and **b,d** OS and DFS in patients receiving oxaliplatin. Hazard ratios are shown with 95 per cent confidence intervals. PM, peritoneal metastases.

Multivariable Cox regression analysis identified pN category, extent of peritoneal metastases and completeness of cytoreduction as independent prognostic factors for OS (*Fig*. 3). The calculated circulating chemotherapy concentration during HIPEC was not associated with adverse effects on survival or disease recurrence.

## Discussion

This study has shown wide variation in intraperitoneal carrier solution volumes in patients with colorectal peritoneal metastases treated with CRS–HIPEC in three tertiary Dutch centres. Variations in carrier solution volume resulted in different calculated intraperitoneal chemotherapy concentrations for both MMC and oxaliplatin. Calculated intraperitoneal chemotherapy concentrations varied tenfold and fourfold for MMC and oxaliplatin respectively.

Postoperative complication, DFS and OS rates were not affected by differences in chemotherapy concentrations, regardless of HIPEC chemotherapy type.

The recently completed PRODIGE 7 trial^15^ (NCT00769405), which compared systemic chemotherapy combined with CRS–HIPEC (oxaliplatin) or systemic chemotherapy with CRS alone, did not find a significant benefit for oxaliplatin‐based HIPEC over CRS alone^16^, ^17^, ^18^. In both groups, median survival was more than 40 months. Interpretation of the results from the PRODIGE 7 study remains difficult because the additional effect of systemic chemotherapy remains to be proven by the CAIRO6 study (NCT02758951). Other recent studies – the Dutch COLOPEC trial (NCT02231086)^19^ and the French PROPHYLOCHIP trial (NCT01226394)^20^ – investigated different aspects of HIPEC treatment, but still failed to resolve the relative contributions made by cytoreductive surgery, hyperthermia, intraperitoneal chemotherapy and perioperative systemic therapy on survival.

The present study aimed to address the potential impact of the intraperitoneal concentration of the chemotherapeutic agent. The findings suggest that concentration differences, within the limits identified, play a minor role in outcome after CRS–HIPEC.

Theoretically, a low concentration might reduce the efficacy of the agent, but no relationship existed between concentration and patient survival. Nor was there any relationship between complications and different calculated chemotherapy concentrations. Several explanations are possible for the lack of association between concentration and survival. If the lowest chemotherapy concentration was above a threshold required to inhibit cell proliferation and cell cycle progression in tumour cells within the given perfusion period, higher concentrations might not impact on survival. The intraperitoneal chemotherapy, as administered in these protocols, might not have added benefit above the other components of CRS–HIPEC, as suggested by the PRODIGE 7 trial. Variable intraperitoneal volumes may also have had a confounding effect^21^. A recent study^22^ with patient‐derived organoids did, however, find that currently used concentrations might be insufficient for complete eradication of all malignant cells. This merits further investigation, as drug concentrations may be critical as new agents are introduced.

Worldwide treatment variation in CRS–HIPEC regimens is well recognized, and standardization can improve outcomes^23^. The American Society of Peritoneal Surface Malignancies has proposed a standardized MMC protocol based on consensus^8^. Despite a willingness to standardize, differences in protocols still exist, as shown in two recent systematic reviews^6^, ^24^. In the Netherlands there is no standardization for the intraperitoneal carrier solution volume. The total carrier solution volume is based on the intraperitoneal volume, whereas the total chemotherapeutic dose is based on BSA, inevitably resulting in wide variations in concentration.

Although drug concentration was not identified as a significant risk factor influencing survival or complications, optimization of chemotherapy concentrations might contribute to standardized treatments, particularly as new agents are introduced.
